# Novel Therapies for Celiac Disease: A Clinical Review Article

**DOI:** 10.7759/cureus.39004

**Published:** 2023-05-14

**Authors:** Haider Ghazanfar, Nismat Javed, Somin Lee, Mohammed Shaban, Dessiree Cordero, Trishna Acherjee, Khushbu Z Hasan, Abhilasha Jyala, Sameer Kandhi, Ali N Hussain, Harish Patel

**Affiliations:** 1 Gastroenterology, BronxCare Health System, New York, USA; 2 Internal Medicine, BronxCare Health System, Icahn School of Medicine at Mount Sinai, New York, USA; 3 Internal Medicine, BronxCare Hospital Center, Icahn School of Medicine at Mount Sinai, New York, USA; 4 Internal Medicine, BronxCare Health System, New York, USA; 5 Internal Medicine, Mohtarma Benazir Bhutto Shaheed Medical College, Mirpur, PAK; 6 Premedical, Baruch College, City University of New York, New York, USA; 7 Medicine/Gastroenterology, BronxCare Health System, New York, USA

**Keywords:** vaccine, monoclonal antibodies, enzymes, antizonulin, clinical features, celiac disease

## Abstract

Celiac disease is emerging as an autoimmune disorder with increasing prevalence and incidence. The mean age of presentation is also increasing with the passage of time. The delay in diagnosis is partly attributable to the asymptomatic state in which most patients present. The diagnosis of the disease is primarily based on biopsy, but serology can also be included for possible screening purposes. Although the primary management strategy is to eliminate gluten from the diet of such patients; however, compliance with the diet and follow-up to detect healing might be difficult to maintain. Therefore, there is a need to investigate further management therapies that can be easily administered and monitored. The aim of the review is to discuss the epidemiology, clinical presentation, and novel therapies being investigated for celiac disease.

## Introduction and background

Celiac disease (CD) is an autoimmune condition triggered by the gluten component in wheat, rye, barley, and related products [1]. In the past, CD presented during infancy with symptoms of life-threatening malabsorption, but nowadays, the mean age of presentation has increased to 10 to 40 years of age, attributable to increased periods of breastfeeding and the late introduction of gluten into the diet [2]. Clinical symptoms vary from diarrhea, steatorrhea, and flatulence to malabsorption-associated complications like anemia, osteopenia, dermatitis, glossitis, and neuropathy. Taavela et al. reported that the severity of clinical symptoms correlates with histological findings related to villous height, crypt depth, and serological titer such as anti-tissue transglutaminase antibody [3,4].

The estimated global prevalence of CD is approximately 1% based on serological testing, whereas studies using biopsy verification along with serological testing have reported an approximate prevalence from 1:70 to 1:300 [1,5]. Large screening studies in Europe and the USA using serological testing have reported a wide range of prevalence from 1:96 to 1:252 [6,7]. CD is often underdiagnosed because asymptomatic cases outnumber symptomatic patients by 7:1 [6]. Recent studies have shown increasing prevalence in the Middle East, India, Northern China, and Northern Africa [8].

CD has an increased incidence among the genetically predisposed population. Increased occurrence is reported among first and second-degree relatives, i.e., siblings (8.9%), followed by children (7.9%) and parents (3.0%) [9]. About 95% to 99% of individuals with CD express at least one of the two major histocompatibility complexes: human leukocyte antigen (HLA) DR3-DQ2 or DR4-DQ8 [10]. Homozygous HLA-DQ2 individuals report an increased incidence of enteropathy-associated T-cell lymphoma as a complication of CD [10].

Variable manifestations and severity of CD delay the diagnosis of the disease [5]. To avoid underdiagnosis and reduce unnecessary screening tests, it is important that clinicians be educated about the characteristics of CD to monitor suspected CD patients more closely and carefully [5].

## Review

Pathogenesis

Strong genetic links contribute to the pathogenesis of CD. Most patients (95%) express at least one of the two major histocompatibility complex (MHC) 2 molecules, namely, HLA-DQ2 and HLA-DQ8, that play a significant part in the genetic predisposition of CD. The absence of these genes may exclude a diagnosis of CD with a very high negative predictive value [11]. The presence of HLA-DQ2 or HLA-DQ8 is significant and attributed to about 40% incidence of CD in individuals positive for the gene [12].

Diagnostics

CD incidence is increasing worldwide with advancements in diagnostic tools. Previously, the diagnosis of CD was mainly based on clinical symptoms and findings such as malabsorptive symptoms. In 1955, introducing intestinal biopsy and intestinal histopathologic findings into the diagnosis of CD brought a significant change in the diagnosis of CD. Around the 1980s, specific and sensitive serum analyses of autoantibodies were introduced, including anti-tissue transglutaminase, endomysial antibodies, anti-deaminated gliadin peptides, and anti-reticulin antibodies [13].

According to the American Gastroenterological Association Institute, the detection and measurement of immunoglobulin A (IgA) antibody against tissue transglutaminase (tTG) is the initial screening test for CD [14]. In untreated patients with an underlying CD, tTG antibodies were highly sensitive (85-95%) and highly specific (95-99%). However, one of the meta-analyses by Silvester et al. found fluctuations in the specificity and sensitivity of this serologic test mostly related to the degree of gluten exposure before the collection of the sample [15].

Another specific serological test to detect CD is anti-endomysial antibodies (EmA). It has absolute specificity (99-100%) and a sensitivity of 95 to 100%. Anti-EmA has been used as a significant value in patients who undergo intestinal biopsy. Some authors even qualify the statistical significance of this EmA serology test as very similar to intestinal biopsy [16,17]. On the other hand, IgA and IgG antibodies against gliadin (gluten’s component protein) have also been linked with a diagnosis of CD but it has relatively lower specificity than other diagnostic serology markers [18].

A serology test of antibodies (IgG and IgA) against reticulin using indirect immunofluorescence assay (IFA) was first described back in 1977; this test detects antibodies against the reticular fibers of the endomysium. There are five different reticular fibers of endomysium, R1 to R5, but only R1 is linked with CD. Even though these antibodies are detected in the target population, they are limited in use because a rodent substrate is needed to perform the test, and very few studies use these IgG and IgA reticulin antibodies as diagnostic tests in comparison with other serologic tests. Therefore, this serum marker is no longer a desired screening test for CD, but this test is still requested by some clinicians as a diagnostic assessment for CD [19].

There is an ongoing debate on the requisition of an intestinal biopsy as confirmatory diagnostic criteria. The European Society for Pediatric Gastroenterology, Hepatology, and Nutrition (ESPGHAN 2020) proposed that patients with elevated serum transglutaminase 2 (TG2) IgA levels >10 times the upper limit of normal do not require a biopsy to diagnose CD. In the USA, this approach has not been adopted and the non-biopsy pathway described in the ESPGHAN guideline was also not implemented in Central Europe Guideline [14]. Although management is multi-disciplinary, the role of a dietitian seems to form the cornerstone of therapy, as discussed in many guidelines [20].

Role of a gluten-free diet

Generally, a gluten-free diet (GFD) is recommended for all patients with CD. Immune stimulation occurs with the ingestion of gluten; therefore, all food components containing gluten and its derivatives must be eliminated from the diet [21]. Although this diet restriction and its compliance are challenging for most patients, adhering to GFD has shown improvement in symptoms and it promotes duodenal mucosa healing [22]. A study by Rubio-Tapia et al. showed mucosal recovery in about 35% and 66% of patients in two to six years after starting a GFD, respectively, with 82% of patients improving symptomatically [23].

Several studies tried to conclude a safe threshold for daily gluten intake. Catassi et al. showed that chronic exposure to small amounts of gliadin can cause a dose-dependent relapse of their symptoms, for example, the group that received 10 mg gliadin per day had minimal mucosal changes in jejunal histopathology whereas another group that consumed 50 mg gliadin per day showed a significant histomorphology mucosal change [24]. However, the debate on the tolerable threshold of gluten ingestion is still controversial.

Different studies showed that in some patients, complete resolution of symptoms and mucosal recovery could not be achieved even with GFD. These GFD non-responders might have gluten contamination or other concomitant pathologies including small-bowel bacterial overgrowth, lactose intolerance, functional bowel disorders, and microscopic colitis [23].

Several studies suggested that a strict GFD may help standard GFD non-responding CD patients. The concept of gluten contamination elimination diet (GCED), strict-GFD, was developed to remove the smallest amounts of gluten from the diet. The strictest dietary regimen focuses on the use of naturally gluten-free products rather than processed gluten-free food. In a study by Hollon et al. on 17 patients who did not respond to original GFD, the patients were started on a GCED and 82% of them were symptom-free after three to six months of GCED [22].

Refractory CD refers to cases still showing continuous clinical symptoms and signs as well as histological evidence of villous atrophy despite adhering to a strict GFD for at least 12 months [22]. Many clinicians recommend maintaining a lifelong GFD for CD patients. Prolonged inflammation and mucosal damage in CD patients can contribute to an increase in lymphoproliferative disorder and can affect overall mortality. The risk of the development of malignancies such as enteropathy-associated T-cell lymphoma (EATL) has been reported. This T-cell lymphoma originated from intraepithelial T-cells of the small intestine, which may result from higher and prolonged exposure to gluten and mucosal inflammation. Cases of small bowel adenocarcinoma are also reported in CD patients, but the exact pathogenesis is still unknown [22,25].

The GFD is still questionable in some patients, so the GFD approach should be targeted and individualized by patients. The data on GCED are limited in the current literature, but it is worth considering in patients who are non-responsive to traditional GFD [21]. In addition to psychological problems, prolonged GFD can cause vitamin B deficiency and therefore, repletion regimens are required [25,26].

Role of genetically modified wheat

Genetically modified wheat (*Triticum aestivum*) has immunotoxic properties that are coded for by chromosomes 1 and 6. Studies have discussed the manipulation of these genes to reduce the immunotoxic components of gluten. One study explored the removal of genes from chromosome 1 that code for β, γ, and ω gliadin fractions and observed that toxicity was attenuated while the mechanical properties of wheat remained intact [27].

Prolamins from another wheat variant were also tested in patients with CD. Although the variant had been formed by the removal of predominantly toxic epitopes in gliadin, the increase in pro-inflammatory cytokines, such as interferon-gamma (IFN-γ) and tumor necrosis factor-alpha (TNF-α), as well as in anti-tTG antibody levels made the substitution unsuitable for patients with CD [28].

The International Wheat Genome Sequencing Consortium has recently delivered a high-quality annotated reference genome sequence of the Chinese spring wheat. The new sequence shows promise in expediting the development of genetically engineered wheat with attenuated immunotoxicity while preserving its gastronomic or agronomic properties [29,30].

Role of thermally modified wheat

Di Luccia et al. studied the detoxification of wheat gluten proteins by the use of microwaves [31,32]. The idea of the technology was to attenuate the immunotoxicity of gluten by 99%. However, the immunotoxicity despite being apparently attenuated using R5 monoclonal antibody method remained unchanged when evaluated using the G12 method [33,34]. Currently, two trials investigating the impact of this gluten-friendly bread are underway.

Results from one trial revealed that Gluten Friendly™ bread did not induce new symptoms in patients and caused uncoupling of mucosal injury. However, the results also showed that uncoupling of mucosal injury was correlated with patients’ baseline microbiota and metabolism [35].

Newer approaches to the management of celiac disease

Recent advances in the understanding of CD have attracted several treatment approaches for CD, for example, decreasing the immunogenic gluten content in crops [36], introducing digestive enzymes and intra-luminal peptidases [37], inhibition of tTG, blockage of HLA-DQ presentation, and silencing of gluten-reactive T cells [38]. Molecular approaches using zonulin antagonists by suppressing the absorption and passage of immunogenic peptides are also under discussion. Induction therapy to increase tolerance to gluten via gluten tolerizing vaccines is also another new approach.

**Figure 1:**
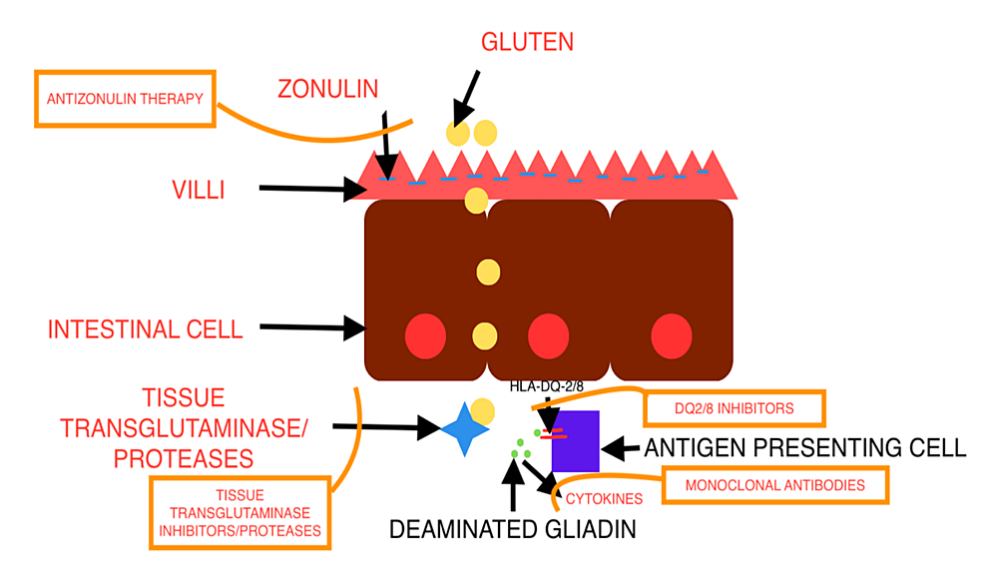
Celiac disease and the targets of novel therapies Image credits: Nismat Javed.

Furthermore, introducing anti-inflammatory drugs and anti-cytokines such as anti-TNF-α and anti-interleukin 15 (IL-15) to CD therapy are also potential promising areas of approaches to CD therapy [37]. These approaches have been summarized in Figure 1.

Polymerizing binders

Polymeric binders can be used for binding gluten and preventing further metabolism, for example, poly(hydroxyethyl methacrylate-co-styrene sulfonate) studied by Liang et al. They observed that after the sequestration of alpha-gliadin, no toxic impact on permeability was observed [39,40]. Another study additionally noted that villous damage had been reduced [41].

Anti-zonulin therapy

Zonulin, a regulator of epithelial permeability at apical tight junctions, is involved in gluten digestion [42]. In the zonulin pathway, gluten products attach to the chemokine receptor CXCR3 on the luminal aspect of the intestinal epithelium, increasing zonulin formation that activates tight junction relaxation through the PAR2/EGFR (protease-activated receptor 2/epithelial growth factor receptor) pathway, increasing permeability of intestinal epithelium and leading to an influx of gliadin [43]. This modulation of the intercellular tight junction by the zonulin pathway has been noted in several chronic and acute inflammatory diseases [44]. Zonulin antagonists would decrease gliadin uptake [36]. One such inhibitor, larazotide acetate (AT-1001), functions as a gut permeability regulator to treat CD and could be a therapeutic strategy in many chronic and acute inflammatory CD [44,45]. Hoilat et al. [45] conducted a meta-analysis of trials to study the efficacy and safety of larazotide acetate (AT-1001) in patients with CD and AT-1001 treatment was significantly correlated with symptomatic improvement after gluten challenge in terms of the CD-specific GI symptom rating scales, especially for gluten-related diarrhea [45].

Tissue transglutaminase inhibitors

Some suggest that TG2 might be one of the earliest disease-relevant proteins encountering immunotoxin gluten in the celiac gut. The transamination and deamination reaction catalyzed by TG2 is essential for the pathogenesis of CD because of the same metabolism for the dietary gluten peptide [46,47]. In CD, deamidation of glutamine residues in gluten peptides stimulates T cells and inflammation cascade that subsequently cause mucosal injury. Therefore, the inhibition of deamidation of gluten peptide by TG2 is a potential pathway treatment for CD [48].

Schuppan et al. [48] conducted a preliminary trial to assess a selective oral TG2 inhibitor, ZED1227. The primary endpoint was the improvement of villus height to crypt depth ratio as a marker of mucosal damage. Secondary endpoints included intraepithelial lymphocyte density, the celiac symptom index score, and the CD questionnaire score for assessment of the health-related quality of life. ZED1227, an oral TG2 inhibitor, significantly improved the ratio of villus height to crypt depth and intraepithelial lymphocyte density. The group receiving a 100-mg dose of ZED1227 showed improved symptoms and quality-of-life scores [48].

Matuchansky suggested the possibility of added mechanisms of action of ZED1227 in CD therapy. TG2-induced degradation of the anti-inflammatory peroxisome proliferator-activated receptor γ is also a key player in the early inflammation of CD. Additionally, TG2 is also associated with an increase in transcellular permeability to intact gliadin peptides through trimeric immune complexes of CD71 (a transferrin receptor), secretory IgA, and transglutaminase [49]. ZED1277, a TG2 inhibitor, can be utilized in CD therapy by inhibiting the degradation of peroxisome proliferator-activated receptor γ and decreasing transcellular permeability, in addition to decreasing T cell immune response in CD patients [46,48,49].

Silencing RNA therapies

Silencing RNA therapies in gelatin-based nanoparticles is also another possible target because they silence human tissue transglutaminase 2 (tTG-2) and IL-15, both of which are involved in the pathogenesis. Results from one study revealed that these particles were internalized in the cytoplasm in two hours and caused a 60% reduction in the gene products of tTG-2 and IL-15 in 72 hours, which decreased both IFN-γ and TNF-α levels [50].

Enzyme therapies

Oral enzyme therapy can also be used to deliver proteolyzing gluten directly to the gastrointestinal tract to detoxify ingested gluten by means of gluten-degrading enzymes, glutenases, mixtures of germinating wheat proteases, or purified prolyl endopeptidases (PEPs) [51,52].

Stenman et al. [53] investigated whether isolated proteases from germinating wheat grain can diminish the gluten effects. They found that protease-pretreated gliadin resulted in less in vitro T cell proliferation and autoantibodies production than with unprocessed gliadin. It was concluded that germinating wheat enzymes reduce the inflammation of wheat gliadin in vitro and ex vivo [53]. Siegel et al. [54] performed a randomized control trial to investigate the safety and tolerability of ALV003, which is a mixture of two proteases that degrade gluten in patients with CD. The authors found that single doses of oral ALV003 were not associated with any severe adverse effects [54].

However, the limitation can be that partially digested gluten protein can still trigger and exacerbate symptoms of CD. Another major challenge of the approach is the susceptibility of this enzyme to the stomach pH. The food composition will influence therapeutic enzyme benefits and gluten degradation in the stomach should be completed as much as possible to prevent and suppress an immune response in the proximal small intestine. Furthermore, the time and point of release of PEPs from enteric capsules fluctuate with food amount and content and intestinal pH [52].

More recently, PEPs from different sources, including *Flavobacterium meningosepticum*, *Myxococcus xanthus*, *Sphingomonas capsulata*, and *Aspergillus niger*, have been extensively studied to overcome this enzyme stability in vivo [52]. Rey et al. [55] found proteolytic components in the carnivorous pitcher plant. This plant metabolizes its prey into small peptides by the action of nepenthesin and neprosin enzymes, which can solubilize gliadin and degrade gliadin into the semi-liquified form even within human gastric pH. The byproduct of these digests can also reduce TG2 conversion rates and suppress T-cell recognition, which further decreases immunologic response against gliadin in CD [55].

DQ2/DQ8 inhibitors

CD has a relatively high prevalence in the western hemisphere affecting ~1% of the white ethnic population [56]. CD has a strong genetic HLA association with HLA-DQ2 and HLA-DQ8 molecules. In genetic susceptibility, the expression of HLA-DQ2 and HLA-DQ8 plays an essential role in inducing an immune response to gliadin protein [57]. HLA-DQ induces the CD4 T cells to recognize antigenic gluten peptides. Recently, HLA-DQ and its involvement in CD8 T cells' immunologic response have drawn attention. A therapeutic approach by blocking or inhibiting this HLA-associated immune response has been discussed [58].

Kapoerchan et al. [59] noted that proline-rich gluten peptides interact with the human HLA-DQ2 molecules and induce immune responses that can lead to CD development. A series of gluten peptides of proline replaced azidoproline residue. This azidoproline residue gluten peptide binds to HLA-DQ2 instead. Azidoproline residue gluten peptides were non-immunogenic and even blocked gluten-induced immune cascade by suppressing CD4+ T-cell response [59].

This therapeutic approach presents unique challenges. Due to its peptide nature, the inhibitors are susceptible to degradation, and a higher concentration is needed for optimal impact [53].

Monoclonal antibodies

IL-15 is a pleiotropic cytokine that stimulates the generation of natural killer (NK), natural killer T (NK-T), γδ, ILC1, and memory CD8 T cells. IL-15 plays pathogenetic roles in organ-specific autoimmune diseases, including CD [60]. Yokoyama et al. [56] utilized trans-genetic mice to express human IL-15 in enterocytes. These mice developed villous atrophy and severe duodenal-jejunal inflammation with a massive accumulation of NK-like CD8+ lymphocytes in human IL-15-expressed enterocytes mucosa [56]. Yokoyama et al. demonstrated that the blockade of IL-15 signaling with an antibody (TM-beta1) against interleukin 2 (IL-2)/IL-15 R beta (CD122) causes a reversal of the autoimmune intestinal damage. These findings support the notion that uncontrolled expression of IL-15 is critical in the pathogenesis of refractory CD [56].

Emerging evidence has implicated a central role of IL-15 in the perpetuation of inflammation and tissue destruction in CD [56]. Diverse approaches are developed to block IL-15 action. IL-15 represents an attractive target for developing new therapies for CD [56].

AMG 714 is the first anti-IL-15 monoclonal antibody to treat CD [61]. Lähdeaho et al. [61] investigated the effects of AMG 714 in patients with CD who underwent gluten challenge in a phase 2a trial. The primary endpoint was the percentage change of the villous height-to-crypt depth (VHCD) ratio. The secondary endpoints were CD3-positive intraepithelial lymphocyte density, clinical symptoms improvement measured by Gastrointestinal Symptom Rating Scale (GSRS), Bristol Stool Form Scale (BSFS), and changes in serology anti-tTG and anti-deamidated gliadin peptide (DGP) antibodies from baseline. Unfortunately, the primary endpoint was not significantly different between the placebo and AMG 714. Though AMG 714 has improved the clinical symptoms, particularly diarrhea. The authors stated that the positive effects on intraepithelial lymphocyte density and symptoms suggest that AMG 714 may be warranted in patients with non-responsive CD, though requiring more randomized control studies [61].

Celiac vaccines

A vaccine based on a set of gluten peptides that can desensitize and increase tolerance to gluten peptides as a part of induction therapy has also drawn attention. In the transgenic HLA-DQ2 mouse models, repeated administration of select immunogenic gliadin peptides suppressed T cell proliferation and decreased pro-inflammatory cytokines, such as IL-2 and IFN-γ [52]. Nexvax2® (ImmuSanT, Cambridge, MA), a desensitizing vaccine consisting of three immunogenic gluten peptides from wheat, barley, and rye, has been developed as an immunotherapeutic and prophylactic agent to restore gluten tolerance [52].

In a phase I clinical trial (NCT00879749), Nexvax2® was administered weekly to HLA-DQ2-positive patients on a strict GFD by intradermal injection for three weeks. Detection of IFN-γ-producing Nexvax2®-specific T cells in several subjects confirmed the bioactivity of the vaccine [62].

In 2017, Goel et al. [63] published the results of the phase 1 trial for the newly developed vaccine for CD, Nexvax2. The promising vaccine includes immunodominant peptide epitopes for CD4 T cells specific for gluten to render them unresponsive to stimulation secondary to gluten exposure. Because of transient, acute GI adverse events with onset two to five hours after initial doses of the vaccine, the maximum tolerated dose of Nexvax2 was 150 μg for twice-weekly intradermal administration over eight weeks. This dose modified immune responsiveness to Nexvax2 peptides without deterioration in duodenal histology. Vomiting, nausea, and headache were evident in about 5% of participants, particularly in 50% of the Nexvax2 150 μg group versus 0% with lower doses. The GI symptoms after the first dose resembled those associated with oral gluten challenge. There was no significant difference in the VHCD ratio in distal duodenal biopsies [63].

Stem cell therapy

Intestinal crypt cells are in a dynamic state of turnover and differentiation, making stem cell therapy targeting markers CD133+/Lgr5+ a promising area of treatment. Autologous stem cell transplant has been studied in patients with refractory CD and results showed a rapid clinical response that lasted for two years [64]. However, the adverse effects of the treatment including neutropenia might limit the therapy to only a few select cases.

## Conclusions

CD, an autoimmune disorder, is becoming a cause of global concern with its increasing prevalence and incidence. The disorder poses multiple challenges given the asymptomatic state of the patients, compliance with a gluten-deficient diet, and multiple pathogenic mechanisms. Therefore, there is a need to investigate the clinicopathological spectrum of the disease, specifically, treatment pathways for novel agents.
